# Glycosylated Chitosan Inhibits Pancreatic Cancer Metastasis by Blocking the Caveolin Signaling Pathway

**DOI:** 10.3390/cancers18091473

**Published:** 2026-05-03

**Authors:** Yong Li, Jacob Paul Adams, Jingxuan Yang, Abigael P. Williams, Min Li, Joanne Tuohy, Wei R. Chen

**Affiliations:** 1 Tianjin Key Laboratory of Cancer Prevention and Therapy, Interventional Therapy Department, Tianjin Medical University Cancer Institute and Hospital, Tianjin 300060, China; 2 Biophotonics Research Laboratory, Center for Interdisciplinary Biomedical Education and Research, College of Mathematics and Science, University of Central Oklahoma, Edmond, OK 73034, USA; 3 Stephenson School of Biomedical Engineering, The University of Oklahoma, Norman, OK 73019, USA; jpadams@ou.edu (J.P.A.);; 4 Hematology-Oncology, Department of Internal Medicine, University of Oklahoma Health Sciences Center, Oklahoma City, OK 73104, USA; jingxuan-yang@ou.edu (J.Y.);; 5 VA-MD College of Veterinary Medicine, Virginia Tech, Blacksburg, VA 24061, USA

**Keywords:** pancreatic cancer, caveolin-1, GC, laser therapy, immunotherapy, metastasis

## Abstract

Background and Objectives: Pancreatic cancer is highly metastatic, and metastasis is the main cause of cancer-related deaths. Therefore, finding methods to inhibit pancreatic cancer metastasis has important clinical value. Methods: *N*-dihydrogalactochitosan (GC) is an immunostimulant that can enhance the anti-tumor immune response to inhibit metastasis in combination with photothermal therapy. Results: Here, we found that GC can inhibit the migration of pancreatic cancer cells through interactions with caveolin-1 (Cav-1). The fluorescence of GC-FITC on the cell surface overlaps with Cav-1-Red, is enhanced by adCav-1, and is weakened by siCav-1, indicating that the localization of GC depends on Cav-1. GC inhibits the migration of malignant cells by blocking the signal transduction of Cav-1 and its downstream molecules. In an orthotopic pancreatic tumor model, GC can inhibit tumor metastasis in tumor-bearing mice. Conclusions: These results demonstrate GC’s capacity to act as a partner in pancreatic cancer therapy and indicate future directions for metastatic cancer therapy.

## Simple Summary

Metastasis, or movement of cancer from its initial tumor location to another location within the body, is the leading cause of cancer-related deaths. As pancreatic cancer is highly metastatic, researchers are interested in finding methods to prevent metastasis in pancreatic cancer to improve the outcomes for patients. In this study, *N*-dihydrogalactochitosan (GC), a type of immune therapy, was used to inhibit metastasis in pancreatic cancer. We sought to investigate how GC may interact with cancer cells and if it could prevent them from metastasizing. Our studies found that GC can prevent the metastasis of pancreatic cancer by interacting with a protein located on the surface of cells known as caveolin-1. While GC does not inhibit the growth of the primary tumor, its ability to hinder metastasis makes it a promising option for combination therapies to treat pancreatic cancer.

## 1. Introduction

Pancreatic cancer is the third leading cause of cancer-related deaths. According to statistics published in 2025, the 5-year overall survival rate for pancreatic cancer is only 13%, the lowest among all cancer types, while the median survival rate is less than one year [1]. Pancreatic cancer usually has a low response rate to conventional radiation and chemotherapy, and the combination of multiple chemotherapeutic or targeted drugs has not yet obtained better therapeutic benefits [2]. One reason for this is that, by the time pancreatic cancer is detected, it has already begun to metastasize, as pancreatic cancer is usually asymptomatic until it is late-stage with metastasis. According to the United States National Cancer Institute’s website, 28% of pancreatic cancer patients have metastases spread to regional lymph nodes at the time of diagnosis, while 51% have distant metastases at the time of diagnosis. The 5-year survival rate for pancreatic cancer patients with regional spread is 16.7%, which drops to only 3.2% when the patient has distant metastases [3]. Therefore, the development of new treatment strategies to inhibit metastasis is the key to effective control of malignant pancreatic cancer.

Caveolin-1 (Cav-1) is a 21–22 kDa protein that is a major component of structures called “caveolae”. Caveolae, which are present in the cell membranes of many types of cells, are flask-shaped, invaginated organelle structures with a size of 50–100 nm. Cav-1 is essential for the formation of caveolae, as cells lacking Cav-1 do not possess caveolae on their cell membranes [4,5]. These pits interact with multiple signaling molecules, such as small GTPases, Src tyrosine kinases, and endothelial nitric oxide synthase. Specifically, Cav-1 has been shown to interact with the transcription factor FoxM1 and the JAK-STAT signaling pathway, both of which influence cell survival and proliferation [6,7]. Therefore, Cav-1, acting as a scaffold of caveolae, plays a variety of roles in the process of organizing or regulating these cell signals [8,9,10]. Caveolae interact with different proteins on the cell surface, such as galectins. Galectins are galactose-binding proteins that bind and crosslink branched N-glycans on glycoproteins [11]. Previously, galectin-3 was used with phosphorylated Cav-1 to stabilize focal adhesion kinase (FAK) and promote cell migration [12]. These three functions, Cav-1’s impact on the formation of caveolae, Cav-1’s interactions with signaling molecules, and Cav-1’s interactions with galectins, make Cav-1 a strong candidate for pancreatic cancer therapy.

Although Cav-1 has shown some tumor suppressive functions in certain human cancers, such as melanoma and breast cancer, previous studies have confirmed that, in pancreatic cancer, Cav-1 is up-regulated and is positively correlated with tumor aggressiveness and poor prognosis [7,13,14,15,16,17,18]. For example, Cav-1 expression is related to tumor survival against radiotherapy, as Cav-1 knockout in pancreatic cancer increases tumor cell sensitivity to radiotherapy and apoptosis [7,19]. Additionally, down-regulation of Cav-1 expression in PC cell lines leads to increased susceptibility to commonly used chemotherapeutic agents, such as gemcitabine and 5-fluorouracil [7]. Other studies have shown that Cav-1’s expression can be regulated in some malignant transformation processes. Furthermore, the regulation of Cav-1 can affect the motility of malignant tumor cells, such as through the subsequent regulation of E-cadherin and vimentin, which impact the migration and invasion of cancer cells [20,21,22,23]. Although relevant studies have not yet reached a consistent conclusion, they all confirm that Cav-1 is implicated in the migration of malignant tumors. Therefore, Cav-1 may be an important target for malignant tumor therapeutic intervention.

*N*-dihydrogalactochitosan, or glycosylated chitosan (GC), is a water-soluble compound first described in 2009, synthesized by linking galactose molecules to the free amino groups of chitosan molecules (CS) [24]. GC is a non-toxic, biodegradable product that seeks to improve the bioavailability and usability of CS in biomedical applications by increasing the water solubility of CS [24]. Past studies have confirmed that GC has immunostimulatory effects and can be used in combination with laser irradiation to treat malignant tumors. This treatment method, known as laser immunotherapy (LIT) or localized ablative immunotherapy (LAIT), was first proposed in 1997 by Chen et al. [25]. LAIT combines local laser irradiation with local injection of an immunostimulant, such as GC, in malignant tumors. While killing tumors, it induces the body’s anti-tumor immune response, and it uses photothermal and immune effects to synergistically kill local and metastatic tumors [26,27]. Studies have confirmed that LAIT is an effective treatment method for metastatic cancer [28,29], and it has been clinically applied in various advanced malignant tumors, such as breast cancer and melanoma, achieving promising results [30,31].

In this study, we found that, in addition to immune stimulation, GC can inhibit the metastasis of pancreatic cancer, while CS, the precursor of GC, has no such function. This study demonstrates how GC interacts with Cav-1, impacting Cav-1-dependent cell migration and decreasing metastases in a murine model of pancreatic carcinoma. This manuscript presents the results of our investigation of the molecular mechanism of GC in inhibiting the metastasis of pancreatic cancer.

## 2. Materials and Methods

### 2.1. Cell Culture

The mouse pancreatic cancer (PC) cell line, Panc02-H7, was derived from Panc02 and displays more aggressive tissue invasion than Panc02 [32]. The human PC cell line, PANC-1, was purchased from ATCC (Manassas, VA, USA). All cell lines were tested and verified to be free of mycoplasma and used after two passages from thawing. Panc02-H7 and PANC-1 cells were cultured with DMEM (GIBCO, Grand Island, NY, USA), supplemented with 10% fetal bovine serum (FBS, ATCC, Manassas, VA, USA), penicillin (100 units/mL), and streptomycin (100 µg/mL) (Sigma, St. Louis, MO, USA). All the cells were cultured in a humidified incubator with 5% CO_2_ and 95% air at 37 °C (NuAire, Plymouth, MN, USA).

### 2.2. Transfections

Panc02-H7 cells were transfected with a red fluorescent protein (RFP) vector control or RFP-tagged Cav-1 using jetPRIME (VWR, Radnor, PA, USA), as recommended by the manufacturer. Gene knockdown experiments were carried out with control siRNA and Cav-1 siRNA at 50 nM using Opti-MEM I Reduced Serum Medium (Life Technologies, Grand Island, NY, USA) in the presence of Lipofectamine RNAiMAX (Life Technologies, Grand Island, NY, USA). The cells were incubated for up to 72 h, validated for target protein knockdown by Western blotting, and processed for subsequent experiments.

### 2.3. Pancreatic Carcinoma Mouse Model

Male and female C57BL/6 mice aged 6–8 weeks were purchased from Harlan Sprague Dawley Co. (Indianapolis, IN, USA). The mice were housed in the animal facility of the Department of Comparative Medicine at the University of Oklahoma Health Sciences Center (OUHSC). All experiments were performed at least 72 h post-delivery (the acclimation period for the OUHSC). All experiments were conducted in compliance with the *Guide for the Care and Use of Laboratory Animals* published by the US National Institutes of Health (NIH) and approved by the OUHSC Institutional Animal Care and Use Committee (IACUC) under protocol #17-032-HCL.

No inclusion criteria were set for this study. The exclusion criteria included the following: recommendations by veterinarians against an animal’s participation and lack of tumor growth following implantation. All criteria were established a priori, and no animal was excluded from this study. Prior to any experimental procedures, twelve mice were randomly assigned to one of three experimental groups (*n* = 4 per group): sham control, CS, or GC. Group sizes were based on previous work related to in vivo GC administration. Randomization was achieved by W.R.C., assigning a treatment group to each mouse without knowledge of the animal’s condition beyond inclusion in this study. Y.L. was aware of the group allocation during experimental procedures, but did not perform the analysis on data collected from these procedures. No other author was aware of group assignments until after all relevant data analysis had occurred.

An orthotopic model of PC was established using a surgical procedure [33]. Briefly, Panc02-H7 (5 × 10^4^ in 50 μL of medium) were injected into the tail of the pancreas of female or male C57BL/6 mice. The animals were ready for experiments in approximately 7–10 days, when the pancreatic tumors reached 8 mm (approximately 300 mm^3^) in diameter. Once the tumors reached a size of 300 mm^3^, they were exposed surgically and intratumorally injected with 0.1 mL of PBS, CS, or GC. The treated mice were monitored daily for signs of morbidity, such as behavioral changes, significant weight loss, and labored breathing.

### 2.4. CS and GC Administration

For in vitro cell treatment, cultured tumor cells were directly incubated with a medium containing CS, GC, or neither. CS was administered in a concentration of 10 μg/mL, while GC was administered in concentrations of 10 μg/mL for all experiments and 50 μg/mL for viability studies.

For in vivo tumor treatment, tumor-bearing mice were anesthetized with 4% isoflurane/96% oxygen using a precision vaporizer. During the treatment, the orthotopic tumors were surgically exposed, and 0.1 mL of GC or CS (50 mg/kg, Immunophotonics Inc., St. Louis, MO, USA) was directly injected intratumorally. The sham control was performed under the same conditions with the injection of 0.1 mL of PBS. The treatments were performed cage by cage, with a random selection order applied to each cage as well as the order of cages treated.

Seven days post-treatment, the mice were euthanized according to approved protocols. The primary tumors were harvested and weighed individually. Additionally, the mesentery of each mouse was collected to quantify metastases; specifically, metastatic growths were counted by a blinded researcher. All tumor weighing and metastasis quantification were performed by a lab member unaware of the group assignments. As an additional clinical endpoint, the mice were euthanized if their tumor volume exceeded 2000 mm^3^. Tumor volume was estimated by length (mm) × width (mm)^2^/2.

### 2.5. Migration Analysis

#### 2.5.1. In Vitro Scratch Assay

Cells cultured in dishes (3 cm, Corning Costar, Lowell, MA, USA) were scratched using a 10 μL Eppendorf tip and washed 3 times with PBS. The cells were then cultured for another 6 h with or without GC (2 µg/mL). Images were analyzed by Image J software (version 1.52k).

#### 2.5.2. Transwell Invasion and Migration Assay

Transwell assays were performed in transwell migration chambers with an 8.0 μm pore size with or without Matrigel (Corning Costar, Lowell, MA, USA). For the invasion assay, cells (1 × 10^5^) in 100 μL of serum-free medium were added to the upper chamber coated with Matrigel. Another 600 μL of medium with 10% FBS and GC or CS was added to the lower chamber. After 24 h, the non-invaded cells in the upper chamber were gently removed with a cotton swab, and the cells attached to the lower surface were fixed with pre-cooled methanol and stained with 0.1% crystal violet. Five fields of each chamber were randomly selected, and the cell numbers were counted under a microscope. For the migration assay, the cells were seeded into upper chambers that were not coated with Matrigel. The following steps in the migration assay were the same as in the invasion assay.

### 2.6. Flow Cytometry

FITC-conjugated GC was prepared using a protocol involving a reaction between the isothiocyanate group of FITC and the primary amino group of GC, as previously described [34]. For GC-FITC staining analysis, treated tumor cells were incubated with GC-FITC for 6 h and then rinsed with PBS 3 times. Trypan blue was used for quenching fluorescence on the cell surface. For cell death analysis, treated cells were stained with Annexin V-FITC and rinsed with PBS 3 times. For endocytosis analysis, treated cells were incubated with 20 nm fluorescent beads for 1 h and then rinsed with PBS 3 times. Data acquisition was performed with a Stratedigm S1200Ex 3 laser flow cytometer (Stratedigm, San Jose, CA, USA), and the FlowJo software (version 10.5) was used for data analysis.

### 2.7. Immunofluorescence

For co-localization analysis of GC and Cav-1, cells transfected with RFP-Cav-1 were grown on cover slips, incubated with FITC-GC for 6 h, fixed in 4% paraformaldehyde (PFA) (Sigma, St. Louis, MO, USA) for 20 min at room temperature, and then washed with PBS. Fluorescence images were acquired using an ultrahigh-resolution microscope, DeltaVision OMX SR, with a Ring-TIRF system (GE Healthcare, Pittsburgh, PA, USA).

For F-actin analysis, treated cells were grown on cover slips, fixed in 4% PFA for 20 min at room temperature, and washed with PBS. The cells were then permeabilized in 0.2% Triton X-100 for 30 min at 4 °C and blocked in 1% BSA for 1 h at room temperature. The fixed cells were incubated with Phalloidin-Alexa Fluor 594 (Thermo Scientific, Waltham, MA, USA) for 1 h at room temperature and then washed with PBS. Finally, cover slips were mounted on microscopy slides using Prolong Gold Antifade Mountant with DAPI (Invitrogen Inc., Eugene, OR, USA). Fluorescence images were acquired using the LSM 510 META confocal system (Carl Zeiss, Thornwood, NY, USA).

For e-cadherin and vimentin expression, individual tumors were fixed in 4% PFA and cut into 5 μm thick sections using a paraffin slicer (Thermo Scientific, Waltham, MA, USA) before staining with H&E and Alexa Fluor 647-conjugated anti-mouse CD324 (e-cadherin) antibody (BioLegend, San Diego, CA, USA) or Alexa Fluor 647-conjugated anti-vimentin antibody (BioLegend, San Diego, CA, USA). The samples were analyzed by fluorescence microscopy (Olympus, Center Valley, PA, USA).

### 2.8. Western Blotting

The following primary mAbs were used: α-actinin rabbit mAb, vinculin rabbit mAb, caveolin-1 rabbit mAb, β-actin mouse mAb (Cell Signaling, Beverly, MA, USA), paxillin rabbit mAb, FAK rabbit mAb, and FAK (phospho Y397) rabbit mAb (Abcam, Cambridge, MA, USA). Protein samples were subjected to SDS-PAGE and transferred to nitrocellulose membranes using standard methods and commercial reagents (Bio-Rad, Hercules, CA, USA). Membranes were blocked in 5% milk/TBST (Tris-buffered saline and Tween 20) and then incubated with primary mAbs overnight at 4 °C. After washing, the membranes were incubated with horseradish peroxidase (-HRP)-conjugated secondary antibodies (Cell Signaling, Beverly, MA, USA). The membranes were incubated with Pierce ECL Western Blotting Substrate (Thermo Scientific, Waltham, MA, USA), and the chemiluminescent signals were detected using the ChemiDoc-It2 imaging system (UVP, Upland, CA, USA). The band densities were quantified using the ImageJ software (version 1.52k). Original images can be found in Supplemental Materials, Figure S1.

### 2.9. Statistical Analysis

Statistical analyses were performed in GraphPad Prism (version 8.0.2). All data were analyzed with a One-Way ANOVA, followed by the Prism-recommended post hoc test (Bonferroni correction). Statistical tests and assumptions were chosen based on industry standards. For all, a *p*-value of < 0.05 was considered significant. *, *p* < 0.05; **, *p* < 0.01; ***, *p* < 0.001; and ****, *p* < 0.0001. Values are expressed as means ± SEM.

## 3. Results and Discussion

### 3.1. GC Inhibits Cell Migration of Metastatic Pancreatic Cancer Cells

**Figure 1:**
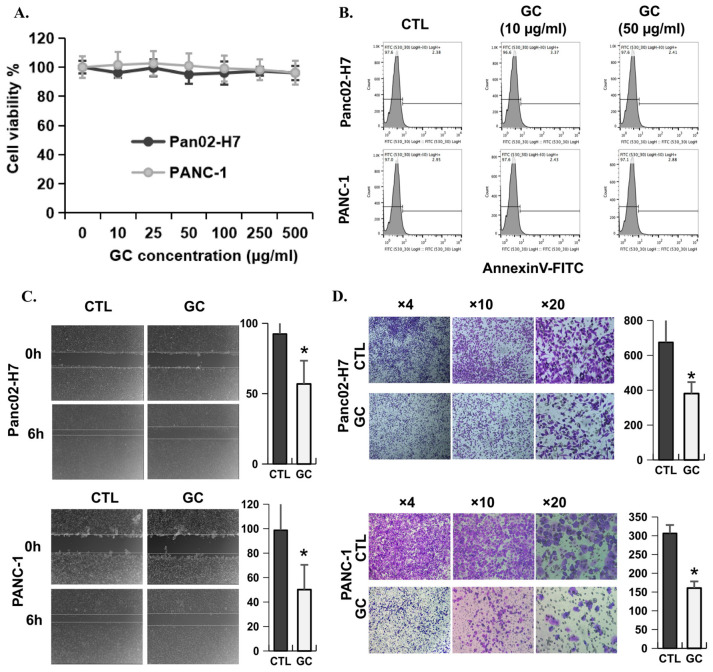
GC inhibits the cell migration of pancreatic cancer cells. (**A**) CCK8 assay and (**B**) flow cytometry staining with Annexin V-FITC showing the viability of Panc02-H7 and PANC-1 cells treated by GC at different concentrations. (**C**) Wound-healing assay (*n* = 18/group; *, *p* < 0.05) and (**D**) transwell assay showing the migration potential of Panc02-H7 and PANC-1 cells treated by 10 μg/mL of GC (*n* = 5/group; *, *p* < 0.05).

GC, an immune stimulator, can significantly inhibit cancer metastasis in animal studies and patient treatment through enhancing antitumor immune response induced by photothermal therapy [25,26,27,28,29,30,31]. However, the direct effect of GC on tumor cells is unclear. To determine whether GC can directly affect the functions of tumor cells, the viability, migration capabilities, and invasion capabilities of metastatic pancreatic cancer cells were investigated. As shown in Figure 1A,B, there were no effects of GC on cell viability and cell apoptosis in both Panc02-H7 cells and PANC-1 cells, as determined by a CCK8 assay and Annexin V staining. However, the wound healing and transwell assays showed that GC significantly (*p* < 0.05) reduced the migration of both Panc02-H7 cells and PANC-1 cells compared with non-treated controls (Figure 1C,D).

**Figure 2:**
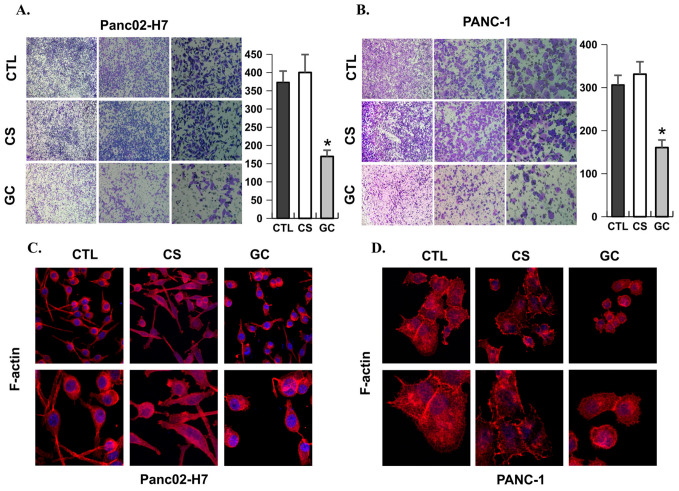
GC inhibits cell invasion and alters migration-related cellular features of metastatic pancreatic cancer cells. Transwell assay showing the invasion potential of (**A**) Panc02-H7 cells (*n* = 5/group; *, *p* < 0.05) and (**B**) PANC-1 cells (*n* = 5/group; *, *p* < 0.05) treated by GC or CS. Fluorescence images showing changes in filopodia-like protrusions by staining F-actin with phalloidin (red) and cell nuclei with DAPI (blue) in (**C**) Panc02-H7 cells and (**D**) PANC-1 cells treated by GC or CS.

As the precursor of GC, chitosan (CS) was used to determine whether the inhibition of GC in tumor cell migration and invasion depends on the structure of CS. The invasion potentials of pancreatic tumor cells under the treatment with GC and CS were investigated and compared using Matrigel-coated transwell assays. As shown in Figure 2A,B, the invasion potentials of both Panc02-H7 cells and PANC-1 cells were reduced by GC. However, there were no changes in the invasion potentials of cells treated by CS. Consistent with these findings, GC-treated tumor cells presented a decrease in filopodia-like protrusions, which aid in cell migration, compared with untreated and CS-treated tumor cells, as shown by the staining of F-actin using fluorescence-labeled phalloidin (Figure 2C,D). Combined, these results indicate that GC can inhibit the invasion and migration of metastatic pancreatic cancer cells. Additionally, our results confirm that GC’s ability to inhibit invasion is absent in its precursor, CS, and suggest that GC’s ability to inhibit migration may also be absent in CS. However, further studies are needed to confirm whether GC, and not CS, possesses the ability to inhibit migration.

Given that most cancer deaths are due to metastasized tumors, GC’s apparent ability to inhibit tumor cell invasion is of great scientific significance. Interestingly, GC does not influence the viability of pancreatic tumor cells, indicating that it needs to be paired with another type of therapy in order to have a robust effect on survival. Furthermore, among CS and GC, GC is uniquely capable of inhibiting tumor cell invasion, suggesting a link between the modified structure of GC (compared with CS) and GC’s inhibitory effects. However, since how GC achieves this effect is still unclear, we next investigated a potential target for this mechanism: Cav-1.

### 3.2. GC Co-Localizes with Cav-1 on the Cell Surface

**Figure 3:**
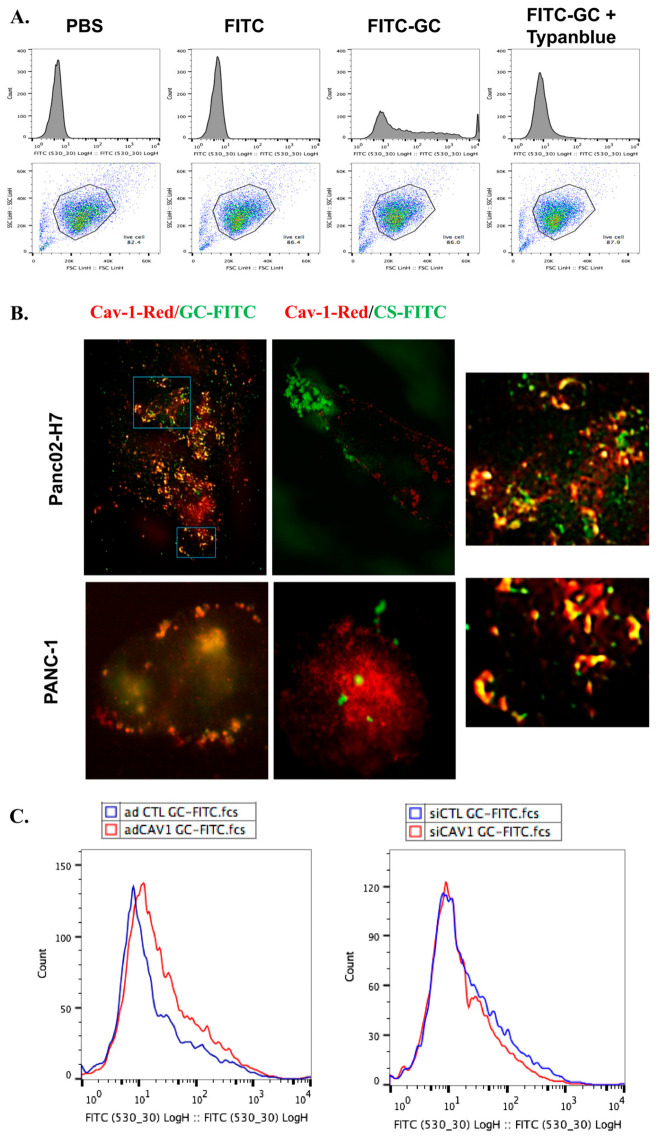
GC co-localized with Cav-1 on the cell surface. (**A**) Flow cytometry analysis showing cell surface staining of GC-FITC. (**B**) Fluorescence images of pancreatic cells. Cells transfected with Cav-1-Red were stained with GC-FITC or CS-FITC and imaged by an ultrahigh-resolution microscope using TIRF. (**C**) Flow cytometry analysis showing surface staining of GC-FITC on cells infected with adCAV-1 or siCAV-1.

A previous study confirmed that GC cannot enter mouse breast tumor cells [35]. Here, we determined whether GC could enter pancreatic tumor cells. Panc02-H7 cells were incubated with FITC-labeled GC and analyzed by flow cytometry. As shown in Figure 3A, Panc02-H7 cells loaded with GC-FITC were quenched by Trypan blue, indicating the localization of GC on the surface of pancreatic cancer cells. Therefore, our hypothesis is that GC inhibits cell migration by interacting with galectins, due to its galactose modifications, and caveolae, due to its inability to be endocytosed. Furthermore, we believe that these interactions lead to GC blocking the functions of Cav-1.

To investigate the interaction of GC with Cav-1, Cav-1-Red transfected tumor cells were incubated with GC-FITC or CS-FITC and observed with an ultrahigh-resolution microscope using the Total Internal Reflection Fluorescence (TIRF) system. As shown in Figure 3B, Cav-1-Red was co-localized with GC-FITC, but not CS-FITC, in both Panc02-H7 cells and PANC-1 cells. As the insets in Figure 3B indicate, GC-FITC was located in the caveolae. To further determine the interactions of GC and Cav-1, Panc02-H7 cells transfected with adenoviral-Cav-1 (adCav-1), which stimulates the production of Cav-1, and small interfering Cav-1 (siCav-1), which inhibits the production of Cav-1, were incubated with GC-FITC and analyzed by flow cytometry. The fluorescence of GC-FITC on the cell surface was enhanced by adCav-1 and reduced by siCav-1, indicating that the surface localization of GC depends on Cav-1 expression (Figure 3C).

Molecules with high expression in tumor cells are of great interest in cancer therapy research, and Cav-1 is of particular interest in this study due to its abundance in pancreatic cancer. We found that Cav-1 and GC co-localize, and that this localization is dependent on the level of expression of Cav-1 in pancreatic cells. Since Cav-1 is highly expressed in pancreatic cancer, and since GC affects the migration of pancreatic cancer cells, our hypothesis is that the interactions between GC and Cav-1 are the key to GC’s effects on pancreatic cancer cell migration. This is clinically relevant for cancers expressing high levels of Cav-1, specifically pancreatic cancer, which is notable for its Cav-1 expression. However, it is unclear whether GC’s localization with Cav-1 affects Cav-1’s functions. Furthermore, these experiments were in vitro, so these results may not translate to in vivo models. Therefore, we investigated GC’s effects on cellular uptake in tumor cells, a known function of Cav-1, as well as GC’s effects on the expression of molecules downstream of Cav-1. Finally, an in vivo study was conducted to further determine GC’s effect on pancreatic tumors in mice.

### 3.3. GC Inhibits Cell Migration Through Cav-1

**Figure 4:**
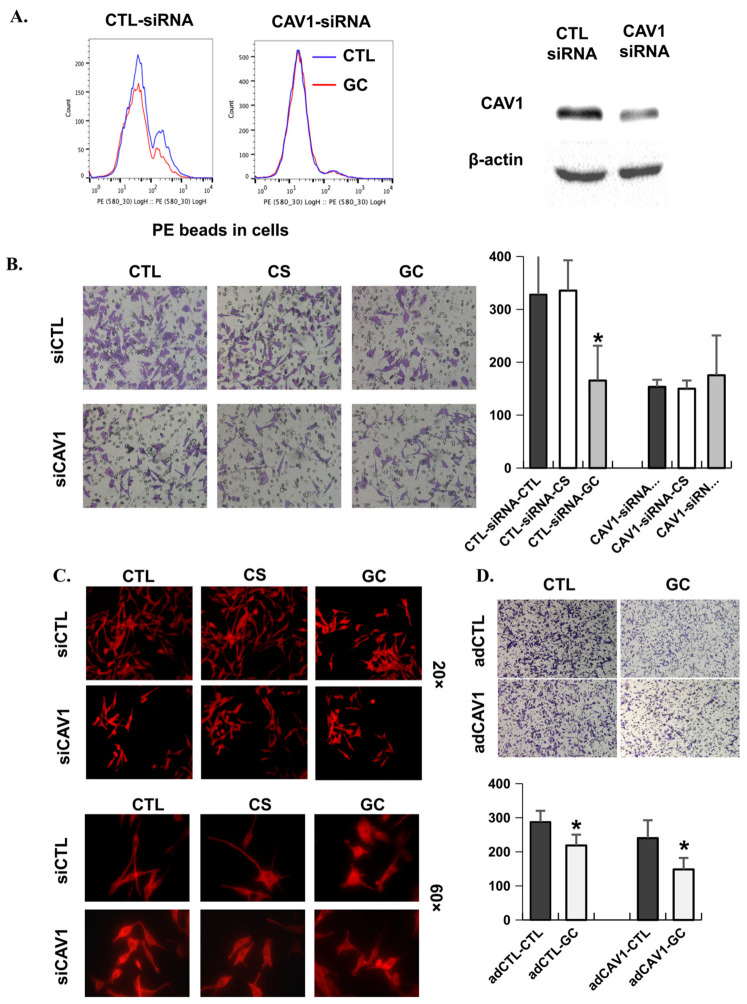
GC inhibits cell migration through Cav-1. (**A**) Flow cytometry analysis showing endocytosis potential of cells treated by GC or transfected with siCAV-1. Western blot showing CAV1 down-expression by CAV1-siRNA. (**B**) Transwell assay showing the metastatic potential of siCAV-1 cells treated by GC or CS (*n* = 5/group; *, *p* < 0.05). (**C**) Fluorescence images showing F-actin in siCAV-1 cells treated by GC or CS. (**D**) Transwell assay showing the metastatic potential of adCAV-1 cells treated by GC (*n* = 5/group; *, *p* < 0.05).

Previous studies investigated whether GC localization affects the functions of Cav-1 by determining the endocytosis potential of tumor cells [36,37]. Panc02-H7 cells transfected with or without siCav-1 were treated with GC and then incubated with fluorescent beads for flow cytometry analysis. As shown in Figure 4A, GC reduced the caveolae-mediated endocytosis (CME) potential of tumor cells. However, when the expression of Cav-1 in the tumor cells was down-regulated by siCav-1, GC could not affect the CME potential of tumor cells. In addition to CME, Cav-1 has also been shown to regulate migration of tumor cells. We investigated how GC affected the invasion and morphology of tumor cells through interactions with Cav-1 and found that GC could not inhibit the invasion and morphology of tumor cells when Cav-1 was down-regulated by siCav-1 (Figure 4B,C). However, when Cav-1 is overexpressed in tumor cells, GC can significantly (*p* < 0.05) inhibit the invasion of the tumor cells (Figure 4D).

**Figure 5:**
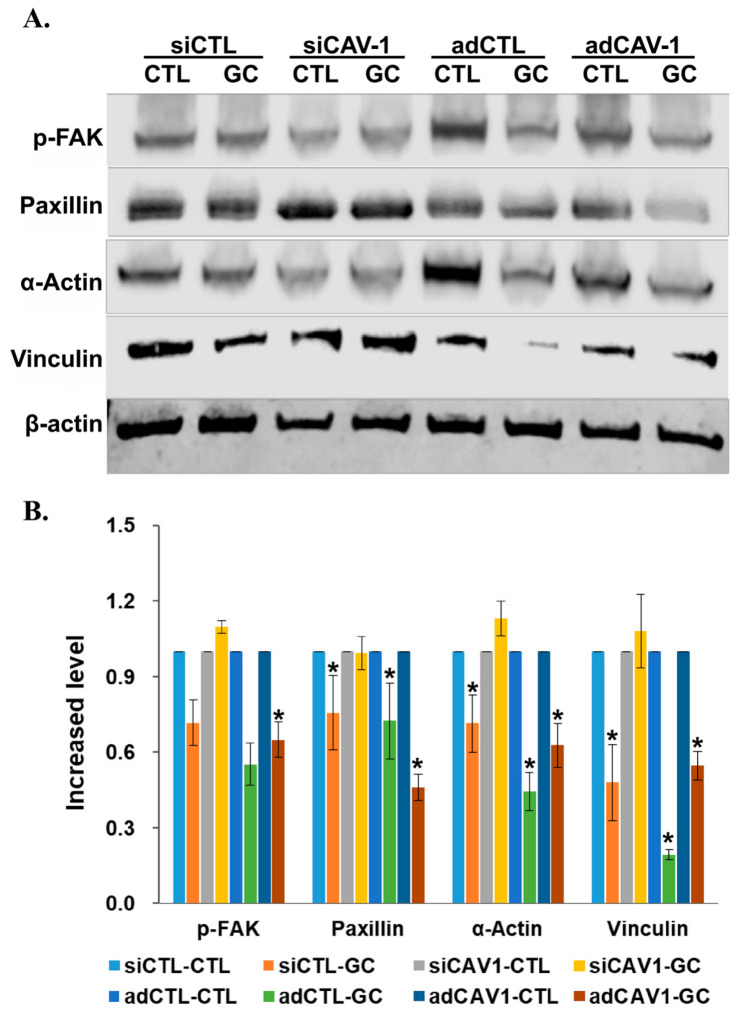
GC affects Cav-1 signaling in pancreatic tumor cells. (**A**) Western blot analysis showing the expression of migration-related proteins in Panc02-H7 cells infected with siCAV-1 or adCAV-1 under the treatment with GC. (**B**) Densitometric analysis of the protein levels in (A) (*, *p* < 0.05).

To further determine whether GC inhibits cell migration by blocking Cav-1 signaling, we investigated the expression of key molecules downstream of Cav-1. As shown in Figure 5, GC treatment can significantly (*p* < 0.05) reduce the expression of p-FAK, paxillin, α-actin, and vinculin, which are proteins downstream of Cav-1. These results indicate that GC inhibits Cav-1-dependent cell migration by blocking the functions of Cav-1 signaling.

**Figure 6:**
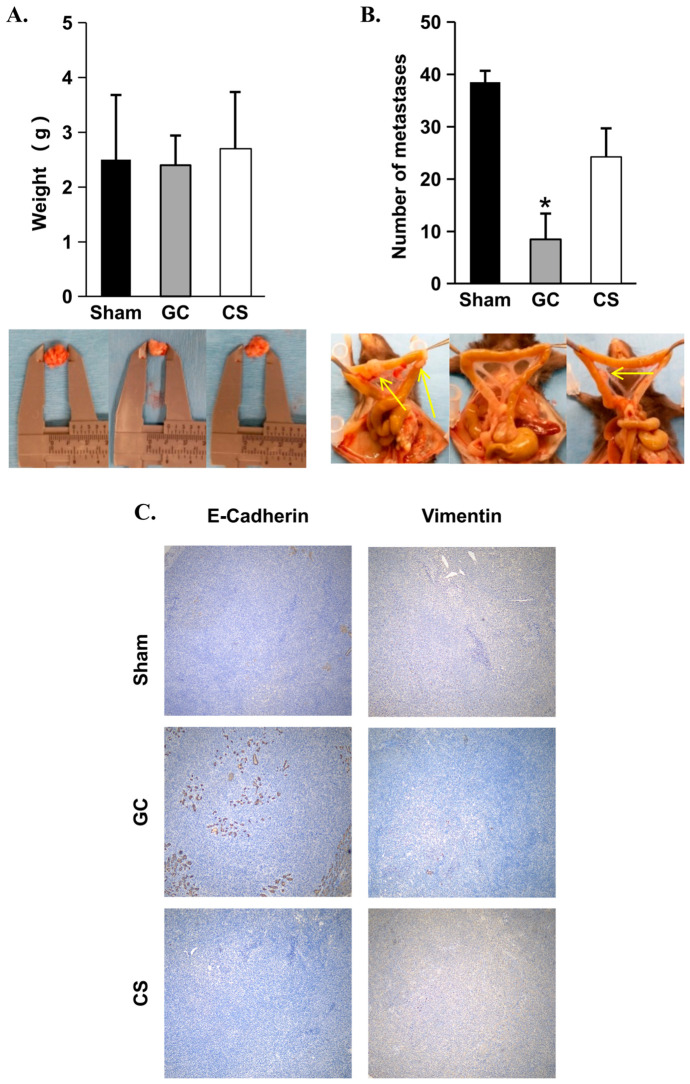
Efficacy of GC on the treatment of orthotopic pancreatic cancer. (**A**) The weight of primary tumors (*n* = 4/group; *, *p* < 0.05) and (**B**) numbers of mesenteric metastases (examples indicated by arrows) 7 days after treatment (*n* = 4/group; *, *p* < 0.05). (**C**) The expression of E-cadherin and vimentin in the treated tumor tissues (*n* = 4/group).

Finally, to determine whether GC can inhibit cancer cell metastasis, Panc02-H7, an aggressive orthotopic metastatic pancreatic tumor model in mice, was used. Compared with the sham control and CS groups, there was no significant difference in primary tumor weight in the GC treatment group (Figure 6A). However, the number of mesenteric metastases was significantly (*p* < 0.05) reduced in the GC treatment group compared with both the sham control and CS groups (Figure 6B). Finally, as shown in Figure 6C, the expression of E-cadherin was significantly (*p* < 0.05) increased, and the expression of vimentin was significantly (*p* < 0.05) decreased in the GC-treated tumors. Previous studies have indicated that the down-regulation of E-cadherin and the up-regulation of vimentin are indicative of metastasis in a variety of cancer types [38,39]. Therefore, these results indicate that while GC treatment cannot inhibit the growth of the primary tumor, it can significantly inhibit metastasis from the primary tumor.

Since Cav-1 and GC co-localize, we investigated how GC affects the functions of Cav-1 and found that GC affects the CME potential of pancreatic tumor cells, the migration of tumor cells, and the expression of molecules downstream of Cav-1. Furthermore, this effect is absent when Cav-1 is down-regulated, indicating that GC requires Cav-1 to produce these effects. We then investigated how GC affects cancer cell metastasis in a mouse model of pancreatic cancer, confirming that GC lacks a direct impact on primary tumors, but it does impact the number of metastases in mice. Additionally, this study confirmed that CS lacks this ability, further indicating the uniqueness of GC. While these results are promising in cancers that have a high expression of Cav-1, GC may not possess the same benefits in other cancer types with a lower Cav-1 expression.

Future studies could investigate whether GC possesses multiple methods of metastasis inhibition. Furthermore, GC cannot effectively treat tumors alone, meaning it will need to be combined with other treatments in order to achieve clinical success. While this manuscript focuses on laser irradiation, previous studies have confirmed that GC can act as a radiosensitizer in breast cancer cells [40]. Investigations into therapies that may synergize with GC, as well as its effects on other cell types, would also help to advance our understanding of GC and its applications for cancer therapy. Finally, a better understanding of the mechanism by which GC enacts its effects on the immune system will prove significant in expanding how it is utilized in clinical applications.

## 4. Conclusions

Metastatic cancers account for a majority of cancer-related deaths, especially in the case of cancers like pancreatic cancer, which are often asymptomatic prior to metastasis. Therefore, therapies that inhibit metastasis are of great clinical significance. GC achieved promising therapeutic effects as an immunostimulant in combination with photothermal therapy of malignant tumors, though its effect on tumor cells, particularly on their migration and metastasis, was unclear. This study investigated a new feature of GC on pancreatic cancer cells: the inhibition of metastasis. Specifically, we found that GC acts on the conduction signal of Cav-1, an important component of the cell membrane structures caveolae, as well as proteins downstream of Cav-1, such as p-FAK, paxillin, α-actin, and vinculin. The reduced expression of these proteins indicates that GC affects Cav-1-dependent cell migration, inhibiting metastasis without directly affecting the viability of pancreatic cancer cells. Furthermore, we showed that CS, a precursor to GC, does not exhibit these effects, highlighting the importance of GC over CS. This study confirms that GC is a strong candidate for combination therapies of metastatic cancers, particularly with ablative therapies like laser irradiation, which directly kill cancer cells. Finally, this study provides direction for future studies, as well as potential clinical applications for GC, in a variety of metastatic cancers.

## Data Availability

The original contributions presented in this study are included in this article. Further inquiries can be directed to the corresponding author.

## Supplementary Materials

The following supporting information can be downloaded at: https://www.mdpi.com/article/10.3390/cancers18091473/s1, Figure S1: Raw images for Western blot (found in Figure 5a), representing (A) p-FAK, the first row in Figure 5a, (B) paxillin, the first second in Figure 5a, (C) α-actin, the first third in Figure 5a, (D) vinculin, the fourth row in Figure 5a, (E) β-actin, the fifth row in Figure 5a, (F) FAK, not shown in final manuscript. Additionally, (G) contains the densitometry readings for each band along with (H) the standard deviation.
