# Supplementary material for: Glycosylated Chitosan Inhibits Pancreatic Cancer Metastasis by Blocking the Caveolin Signaling Pathway
Source: Cancers (Basel). 2026 May 3;18(9):1473. doi: 10.3390/cancers18091473 (PMC13162701; doi:10.3390/cancers18091473)
Supplement: Supplementary file 1 [file cancers-18-01473-s001.zip › cancers-4248302-supplementary.pdf]

Figure S1. Raw images for Western blot (found in Figure 5a), representing **(A)** p-FAK, the first row in Figure 5a, **(B)** paxillin, the first second in Figure 5a, **(C)** α -actin, the first third in Figure 5a, **(D)** vinculin, the fourth row in Figure 5a, **(E)** β -actin, the fifth row in Figure 5a, **(F)** FAK, not shown in final manuscript. Additionally, **(G)** contains the densitometry readings for each band along with **(H)** the standard deviation.

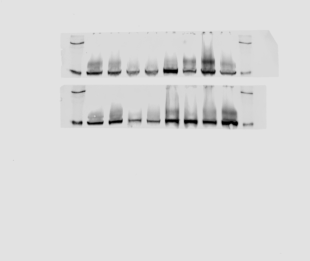

(A)

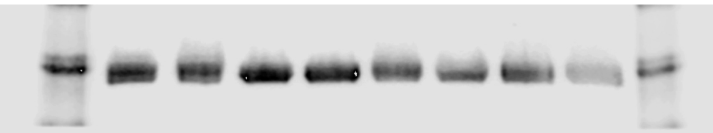

(B)

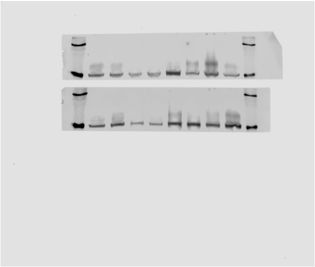

(C)

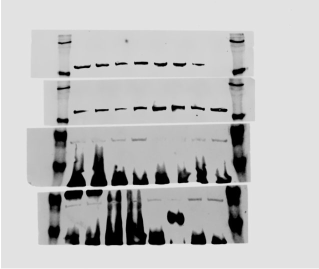

(D)

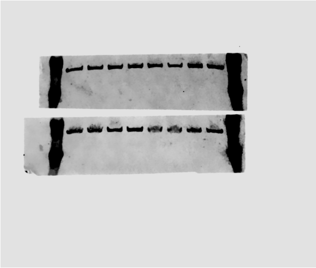

(E)

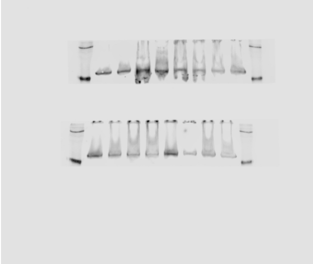

(F)

(G)

Densitometry Readings	p-FAK	FAK	Paxillin	α-actin	Vinculin
siCTL-CTL	1.0000000	1.0000000	1.0000000	1.0000000	1.0000000
siCTL-GC	0.7165770	0.6039277	0.7561716	0.7152505	0.4793945
siCAV1-CTL	1.0000000	1.0000000	1.0000000	1.0000000	1.0000000
siCAV1-GC	1.0963476	0.9024126	0.9936485	1.1306393	1.0802009
adCTL-CTL	1.0000000	1.0000000	1.0000000	1.0000000	1.0000000
adCTL-GC	0.5516208	0.2156993	0.7243860	0.4438890	0.1933681
adCAV1-CTL	1.0000000	1.0000000	1.0000000	1.0000000	1.0000000
adCAV1-GC	0.6494913	0.5057626	0.4616257	0.6269656	0.5464719

(H)

SD	p-FAK	FAK	Paxillin	α-actin	Vinculin
siCTL-CTL	0	0	0	0	0
siCTL-GC	0.089802898	0.0931401	0.147077428	0.113881433	0.150921874
siCAV1-CTL	0	0	0	0	0
siCAV1-GC	0.025111670	0.1155156	0.065447113	0.067271929	0.146107947
adCTL-CTL	0	0	0	0	0
adCTL-GC	0.084171330	0.0345655	0.150971628	0.076493454	0.019442947
adCAV1-CTL	0	0	0	0	0
adCAV1-GC	0.070142837	0.0398627	0.051520742	0.085871555	0.055855943
